# Distinct patterns of diversity, population structure and evolution in the AMA1 genes of sympatric *Plasmodium falciparum* and *Plasmodium vivax* populations of Papua New Guinea from an area of similarly high transmission

**DOI:** 10.1186/1475-2875-13-233

**Published:** 2014-06-14

**Authors:** Alicia Arnott, Johanna Wapling, Ivo Mueller, Paul A Ramsland, Peter M Siba, John C Reeder, Alyssa E Barry

**Affiliations:** 1 Centre for Biomedical Research, Burnet Institute, Melbourne, Australia; 2 Barcelona Centre for International Health Research, Barcelona, Spain; 3 Division of Infection and Immunity, Walter and Eliza Hall Institute of Medical Research, Melbourne, Australia; 4 Department of Medical Biology, University of Melbourne, Parkville, Australia; 5 Department of Immunology, Monash University, Melbourne, Australia; 6 Department of Surgery Austin Health, University of Melbourne, Heidelberg, Australia; 7 School of Biomedical Sciences, CHIRI Biosciences, Faculty of Health Sciences, Curtin University, Perth, Australia; 8 Papua New Guinea Institute for Medical Research, Goroka, Papua New Guinea; 9 Centre for Population Health, Burnet Institute, Melbourne, Australia; 10 Department of Epidemiology and Preventative Medicine, Monash University, Melbourne, Australia; 11 Current affiliations: Division of Infection and Immunity, Walter and Eliza Hall Institute of Medical Research, Melbourne, Australia; 12 Current affiliations: Department of Medical Biology, University of Melbourne, Parkville, Australia

**Keywords:** *Plasmodium falciparum*, *Plasmodium vivax*, AMA1, Malaria vaccine, Population genetics, Papua New Guinea

## Abstract

**Background:**

As *Plasmodium falciparum* and *Plasmodium vivax* co-exist in most malaria-endemic regions outside sub-Saharan Africa, malaria control strategies in these areas must target both species in order to succeed. Population genetic analyses can predict the effectiveness of interventions including vaccines, by providing insight into patterns of diversity and evolution. The aim of this study was to investigate the population genetics of leading malaria vaccine candidate AMA1 in sympatric *P. falciparum* and *P. vivax* populations of Papua New Guinea (PNG), an area of similarly high prevalence (*Pf* = 22.3 to 38.8%, *Pv* = 15.3 to 31.8%).

**Methods:**

A total of 72 *Pfama1* and 102 *Pvama1* sequences were collected from two distinct areas, Madang and Wosera, on the highly endemic PNG north coast.

**Results:**

Despite a greater number of polymorphic sites in the AMA1 genes of *P. falciparum* (Madang = 52; Wosera = 56) compared to *P. vivax* (Madang = 36, Wosera = 34), the number of AMA1 haplotypes, haplotype diversity (*Hd*) and recombination (*R*) was far lower for *P. falciparum* (Madang = 12, Wosera = 20; *Hd* ≤0.92, *R* ≤45.8) than for *P. vivax* (Madang = 50, Wosera = 38; *Hd* = 0.99, *R =* ≤70.9). Balancing selection was detected only within domain I of AMA1 for *P. vivax*, and in both domains I and III for *P. falciparum*.

**Conclusions:**

Higher diversity in the genes encoding *P. vivax* AMA1 than in *P. falciparum* AMA1 in this highly endemic area has important implications for development of AMA1-based vaccines in PNG and beyond. These results also suggest a smaller effective population size of *P. falciparum* compared to *P. vivax,* a finding that warrants further investigation. Differing patterns of selection on the AMA1 genes indicate that critical antigenic sites may differ between the species, highlighting the need for independent investigations of these two leading vaccine candidates.

## Background

Although greater morbidity and mortality from malaria is attributed to infection with *Plasmodium falciparum*, *Plasmodium vivax* is responsible for much of the global malaria burden. This species can also cause severe disease [1-4], has a broader geographic distribution than *P. falciparum* and is increasingly being recognized as less responsive to malaria control measures [5]. Indeed, in the context of malaria elimination, it is thought that the relatively resilient *P. vivax* will present the ultimate challenge in areas in areas of co-endemicity [6].

The greater resilience of *P. vivax* is thought to be predominantly due to its ability to form dormant liver stages [7] and may also be because *P. vivax* is a more genetically diverse parasite than *P. falciparum*. The greater diversity of *P. vivax* has been demonstrated using a panel of global isolates [8] and in an endemic area of Cambodia with similarly low transmission of both species [9,10]. Diversity is an indicator of the evolutionary fitness of a parasite population as high genetic diversity provides greater potential for adaptation to changing environmental conditions and for immune escape, which is facilitated by antigenic polymorphism [11]. *Plasmodium falciparum* diversity is strongly linked with the overall transmission intensity of the broad geographic area [12] and is consistent with decreasing diversity with distance from an African origin [13]. However, diverse *P. vivax* populations have been observed even in very low transmission settings [14,15] and the origin of *P. vivax* is still being debated [16-18]. Defining the genetic diversity of *P. falciparum* and *P. vivax* parasite populations can therefore provide important insight into malaria epidemiology and parasite evolution, in addition to how well the local parasite population might respond to interventions such as malaria vaccines [19].

A broadly effective malaria vaccine is considered the most sustainable approach to controlling and eventually eliminating malaria [20]. One of the major barriers to the development of malaria vaccines is the extreme diversity of leading candidate antigens. Therefore, a better understanding of parasite antigenic diversity is urgently needed [21,22]. As *P. falciparum* and *P. vivax* co-exist in many malaria-endemic regions, it is essential that both species be targeted if elimination is to be achieved. Whilst many leading malaria vaccine candidates are orthologous, on account of biological differences that exist between the species it is not possible to extrapolate findings from one species to the other [8,23-25]. Therefore, the antigenic diversity of leading candidates must be investigated independently for each species.

One of the most promising vaccine candidates for both *P. falciparum* and *P. vivax* is the apical membrane antigen 1 (AMA1) [26-33]. Although important for merozoite invasion, the precise biological function of AMA1 was largely unknown until it was recently reported that *P. falciparum* AMA1 (*Pf*AMA1) is essential for invasion of host cells as it has a direct or indirect role in resealing of the red blood cell at the posterior end of the invasion event [34]. That AMA1 sequences are highly conserved amongst all parasites of the Apicomplexa phylum suggests conservation of fundamental biological properties [35]. Additionally, both the *P. falciparum *[36-38] and *P. vivax *[39-42] AMA1 ectodomains are highly immunogenic. *Pf*AMA1 elicits antibodies that can inhibit invasion of host cells *in vitro *[43-45], thereby contributing to protective immunity in humans naturally exposed to malaria [46,47]. AMA1 is, therefore, considered to be a prime vaccine candidate for both *P. falciparum* and *P. vivax,* however few studies have investigated the genetic diversity of AMA1 in sympatric *P. falciparum* and *P. vivax* populations [48,49]. In Venezuela, the genes encoding *P. vivax* AMA1 (*Pv*AMA1) were reported to be significantly more diverse than *Pf*AMA1. However, *P. vivax* has a much higher prevalence than *P. falciparum* in this region, which may explain the patterns observed [48]. The diversity of each species at the *ama1* locus is yet to be compared in an area highly endemic for both species.

Intense year-round transmission of both *P. falciparum* and *P. vivax* occurs on the north coast of Papua New Guinea (PNG) [50]. Separate population genetic studies of AMA1 have been done in this area for *P. falciparum *[21,35] and *P. vivax *[51,52] however the results cannot be directly compared on account of samples being collected at different time points and because only partial *ama1* sequences were analysed for *P. falciparum*. The findings of these previous studies revealed that PNG parasites have a genetically distinct repertoire of *P. vivax* AMA1 alleles compared to other populations [51], whereas *P. falciparum* AMA1 domain I (DI) sequences collected in the year 2000 from a single, highly diverse parasite population [35] were found to be representative of the worldwide diversity [21].

The aim of this study was to investigate patterns of diversity, population structure and evolution of full-length *P. falciparum* and *P. vivax* AMA1 genes in sympatric parasite populations in two geographically distinct areas of PNG. Analysis of the data confirmed and extended previous findings, revealing important differences in the population biology of *P. falciparum* and *P. vivax* in PNG which may have important implications for the design of AMA1-based vaccines.

## Methods

### Study sites and isolates

The Madang and East Sepik Provinces on the north coast of PNG are areas of intense perennial malaria transmission and have been the focus of malaria research and control efforts for more than half a century. Cross-sectional malaria surveys including asymptomatic volunteers of all ages were conducted in 2005 and 2006 in four catchment areas including Mugil, Malala and Utu in the Madang Province, and the Wosera district in the East Sepik Province. The study sites and parasite isolates have been described in detail elsewhere [53-55]. *Plasmodium falciparum-* and *P. vivax-* infected samples were identified by PCR-based methods [54,55]. The prevalence of both species was found to be similarly high at 22.3 to 38.8% for *P. falciparum *[55] and 15.3 to 31.8% for *P. vivax *[54]. Isolates were also genotyped at highly polymorphic loci to identify monoclonal infections as previously reported [53,54]. A total of 76 monoclonal *P. falciparum *[53] and 102 monoclonal *P. vivax* isolates [54] were selected for analysis in this study. Using microsatellite markers, a moderate to high degree of population structure for *P. falciparum* (i.e. high diversity between populations [53]) but limited geographic population structure of *P. vivax* was identified in this region (i.e. limited diversity between populations [56]). Therefore, for *P. falciparum* only isolates from Mugil were used, and due to the relatively small number of *P. vivax* samples available from each of the three Madang catchments, isolates were combined to form a single population for analysis. Catchment populations were also analysed separately to confirm the patterns observed. The two distinct parasite populations will be referred to as Wosera and Madang throughout the manuscript.

Ethical approval to conduct this study was granted by the PNG Institute of Medical Research Institutional Review Board (Nos 08–08 and 11–05), the Medical Research Advisory Committee of PNG (Nos 10.23 and 11–06), the Alfred Hospital Research and Ethics Unit (No 30/06Q and 420–10) and the Walter and Eliza Hall Institute Human Research Ethics Committee (No 11–09 and 11–12).

### PCR and sequencing

Whole genome amplification (WGA) was performed for all *P. falciparum* samples using the Illustra GenomiPhi V2 Amplification kit (GE Healthcare, NSW, Australia), as per the manufacturer’s instructions. The multiple displacement amplification (MDA) WGA method used has been shown to result in a higher yield of non-artifact DNA templates and reduced amplification bias compared with PCR-based WGA methods [57]. Previously, to investigate whether artifacts were introduced as a result of WGA, *P. vivax* sequences obtained from undiluted genomic DNA and sequences amplified from WGA template were compared [54]. Consistent with previous reports [58,59], results were concordant between the WGA and unamplified DNA. Nucleotides 38 to 1,674 bp of the 1,869 bp *Pfama1* coding sequence, encompassing the prosequence, signal sequence and the complete ectodomain (DI to DIII) were amplified using a modified version of a previously described nested PCR strategy [60,61]. Modifications included: both primary PCR primers Fex (5'-ATGTACTTGTTATAAATTGTAC–3' (Fwd)) and Rex (5'–CAGCTTCTCTTTTATGCTAA–3' (Rev)). For the nested PCR, the published forward primer F2 [60] was used with the TMr reverse primer (5'-GCTGTCGCTGTATTAGCAACTA-3'). As the *P. falciparum* genome is extremely AT-rich, if PCR was unsuccessful, the PCR enhancers, dimethyl sulphoxide (DMSO, 5% PCR grade; Sigma-Aldrich, MO, USA), Betaine (0.05 M PCR grade; Sigma-Aldrich, MO, USA), bovine serum albumin (BSA, 0.1 mg/mL PCR grade; Roche, GmbH, Germany) or polyethylene glycol (PEG6000, 1.5%; Sigma-Aldrich, MO, USA) were added to the PCR mastermix. Sequencing reactions were performed by a contract sequencing facility using the ABI BigDye Terminator Cycle Sequencing kit on an ABI 3730XL automatic DNA Analyser (Macrogen, Seoul, Korea). For *P. vivax*, nucleotides 1 to 1,524 bp of the 1,686 bp *Pvama1* coding sequence, encompassing the signal sequence and the complete ectodomain (DI to DIII) were amplified and sequenced previously as described [48,51,62].

### Analysis

The length of the *Pfama1* ectodomain sequence analysed was 1,335 bp (from nucleotide 300 to 1,635, relative to the reference sequence *3D7* [GenBank accession number XM_001347979.1]) and for *Pvama1,* 1320 bp (from nucleotide 300 to 1620, relative to the reference sequence *Sal-1* [GenBank accession number AF063138]). Differences in length were due to the insertion of five additional residues within *Pfama1* DIII (positions 471 to 473, relative to the reference sequence *3D7*) and the transmembrane region (positions 535 and 540, relative to the reference sequence *3D7*). For each of the *P. falciparum* field isolates, raw sequence data were edited and high quality sequences assembled into contigs using Sequencher version 5.0 [63]. Editing of raw sequence data included trimming the poor quality ends of sequence reads and assembling sequences to the relevant reference sequence in order to generate a consensus sequence. Once assembled to the reference, ambiguous base calls were clarified and if the quality of the chromatogram was not sufficient to enable ambiguous bases to be accurately called, the sample was re-amplified and re-sequenced. Only high quality data was included in the final dataset. Single nucleotide polymorphisms (SNPs) were identified by comparing the consensus sequence for each isolate to the reference strains (*P. falciparum* 3D7). SNPs were confirmed if they were present in at least one other isolate. Rare SNPs, found in only one isolate, were confirmed by amplifying and sequencing a second independent PCR product. Sequences were deposited in GenBank [KF698984 to KF699059 (*Pfama1*) and KC702402 to KC702503 (*Pvama1*)] [51].

Although analysis of the *P. vivax* sequences was previously reported [51], the genomic region investigated in the present study was smaller (1,320 bp) and included an additional 96 bp at the 3' end not analysed previously in order to enable direct comparison with *Pfama1* sequences. Hence population genetic analyses were repeated and extended compared to those performed previously for all *Pvama1* sequences included in the present study. Nine additional reference sequences were also analysed including the *Pfama1* sequence of the *FVO* isolate (GenBank accession no AJ277646.1); six primate-adapted *P. vivax* isolates used for vaccine research (*Chesson I, Belem, India VII, Indonesia XIX, Palo Alto* and *North Korea*; GenBank accession nos EU395587 to EU395593) [64].

Multiple alignments were performed using the *MUSCLE* algorithm implemented in *MEGA* version 5.0 software [65]. Polymorphism and diversity was estimated using *DnaSP* version 5.0 [66] by calculating the total number of polymorphic sites (*S*); synonymous (SP) and non-synonymous (NS) SNPs, average pairwise nucleotide diversity (*π*), number of haplotypes (*h*) and haplotype diversity (*Hd*), which is an allele frequency based statistic analogous to the heterozygosity and is calculated as follows:

$$\mathrm{Hd}=[n/(n-1)]\;\left[1-\sum \left(f_{i}\right)\left({}^{2}\right)\right]$$

Where n is the sample size and *f* is the frequency of the *i*^th^ allele [67]. As the number of haplotypes is influenced by sample size, the allelic richness (*R*_*S*_) was also calculated, as *R*_*S*_ is normalized on the basis of the smallest sample size [68]. *R*_*S*_ was calculated using FSTAT version 2.9.3 [69]. Additionally, allele frequencies of all amino acid polymorphisms were calculated using *CONVERT*, version 1.31 [70].

To identify departures from neutrality, a sliding window analysis of Tajima’s D was performed using *DnaSP* version 5.0 [66]. Negative values of Tajima’s D indicate an excess of rare alleles consistent with directional selection or recent population expansion whereas positive values indicate an excess of intermediate frequency mutations, suggestive of a recent population bottleneck or balancing selection [71]. Balancing selection maintains alleles at balanced frequencies within populations in order to maintain genetic diversity and therefore an evolutionary advantage under immune pressure.

To estimate the amount of recombination in AMA1 genes, the recombination parameter (*R*), based on the variance between the average number of nucleotide differences between pairs of sequences, was calculated using *DnaSP* version 5.0 as follows:

R=4Nr

Where *N* is the population size and *r* is the recombination rate per sequence [72]. All parameters of the equation were estimated from the input data by *DnaSP* software in the process of calculating *R*.

To measure the degree of non-random association between alleles at two or more sites (linkage disequilibrium, LD), *D’ *[73] and *r*^*2*^ [74] were calculated using the ‘Full Matrix LD’ option of *TASSEL* software, version 3.0.157 [75]. LD was calculated only for SP and NS SNPs with a minor allele frequency (MAF) or combined MAF ≥0.10 (‘common’ polymorphisms). ‘Combined MAF’ refers to the combined frequencies of the minor alleles detected at specific sites. Only ‘common’ SP and NS SNPs with a MAF or combined MAF ≥0.10 were analysed as inclusion of rare alleles can artificially inflate values of *D’ *[76]. Tri-allelic SNPs were split so that the major allele was analysed separately with each of the minor alleles. The Fisher’s Exact test was used to measure significance of any associations.

The *Pv*AMA1 model was generated as previously described [51]. The *Pf*AMA1 model was generated using the same protocol [51] with the following modifications. The chimeric template was generated using overlays of the *P. vivax* (Protein Data Bank ID: 1W81) and *P. falciparum* (Protein Data Bank ID: 1Z40) AMA1 crystal structures, and grafting *Pv*AMA1 loop residues onto the *Pf*AMA1 core. The grafted residues were as follows: *Pv*AMA1 residues 41 to 52 (numbering relative to the *P. vivax* reference strain *Sal-1* AMA1 sequence) which correspond to *Pf*AMA1 residues 96 to 107 (numbering relative to the *P. falciparum* reference strain *3D7* AMA1 sequence); *Pv*AMA1 residues 116 to 123 which correspond to *Pf*AMA1 residues 171 to 178; and as the crystal structure of *Pf*AMA1 DIII is yet to be solved, *Pv*AMA1 residues 383 to 400 and 416 to 474, which correspond to *Pf*AMA1 residues 438 to 455 and 471 to 532, respectively. Three *Pf*AMA1 loop structures missing from both the *Pv*AMA1 and *Pf*AMA1 crystal structures were generated automatically by Modeller using only stereochemical and geometric restraints: *Pf*AMA1 residues 264 to 274 (loop 1), 383 to 387 (loop 2) and 456 to 470 (loop 3). The *P. falciparum 3D7* reference sequence (GenBank accession no XM_001347979.1) was aligned against the chimeric template sequence in order to generate the *Pf*AMA1 model. As the incomplete *Pf*AMA1 crystal structure necessitated modeling three missing loop structures and grafting the entire DIII from the *Pv*AMA1 crystal structure, one hundred *Pf*AMA1 models were generated in order to determine the optimized model with the lowest final probability density function (PDF) energy for structural analysis. Discovery Studio, version 3.1 (Accelrys, San Diego, CA, USA) was used to prepare figures.

## Results

### Polymorphism and diversity of *Plasmodium falciparum* and *Plasmodium vivax* AMA1 genes

The total number of polymorphic sites (S) in *Pfama1* was almost double that of *Pvama1* (Table 1). Similarly, the number of NS SNPs for *Pfama1* was nearly double that of *Pvama1* (Table 1). Conversely, the number of SP SNPs was much lower in *Pfama1* than for *Pvama1*. Despite fewer polymorphic sites overall, the number of *Pvama1* haplotypes was more than four times that of *Pfama1* in Madang and nearly double the number detected for *Pfama1* in the Wosera. Haplotype diversity was very high for both species, with *Pvama1* haplotype diversity close to the maximum of 1, indicating that all haplotypes are rare (Table 1).

**Table 1:** **Estimates of AMA1 genetic diversity for *P. falciparum *and *P. vivax *in PNG**

		***P. falciparum***		***P. vivax***	
		**Wosera**	**Madang**	**Wosera**	**Madang**
Whole ectodomain	*n*	44	32	41	61
	*S*	56	52	34	36
	π (x 10^-3^)	14.2	13.4	7.6	8.5
	NS	48^a^	47^b^	26	29^c^
	SP	1^a^	1^b^	8	4^c^
	*h*	20	12	38	50
	*Hd*	0.92	0.91	0.99	0.99
	*R*_*S*_	19	12	38	45
	*R*	45.8	36.6	70.9	66.2
DI	*S*	36	32	21	23
	π (x 10^-3^)	25.1	24.5	15.7	17.3
	NS	30^a^	29^b^	14	16^c^
	SP	1^a^	1^b^	7	4^c^
	*h*	18	12	21	25
	*Hd*	0.92	0.91	0.96	0.95
DII	*S*	8	8	4	6
	π (x 10^-3^)	8.3	6.6	2.3	2.6
	NS	8	8	4	6
	SP	0	0	0	0
	*h*	10	10	5	9
	*Hd*	0.86	0.85	0.59	0.57
DIII	*S*	7	7	1	1
	π (x 10^-3^)	14.8	13.7	0.2	1.3
	NS	7	7	1	1
	SP	0	0	0	0
	*h*	9	8	2	2
	*Hd*	0.86	0.84	0.04	0.25

DI=domain I; DII=domain II; DIII; domain III; *n*=number of samples; *S*=number of polymorphic sites; π=nucleotide diversity; NS= number of non-synonymous single nucleotide polymorphisms (SNPs); SP=number of synonymous SNPs; *h*=number of haplotypes; *Hd*=haplotype diversity; *R*_*S*_: allelic richness; *R*: recombination parameter.

^a^Complex codons not analysed by DnaSP software: 3 codons (292, 293, 294) (301, 302, 303) (304, 305, 306).

^b^Complex codons not analysed by DnaSP software: 2 codons (292, 293, 294) (301, 302, 303).

^c^Complex codon not analysed by DnaSP software: 1 codon (550, 551, 552).

To ensure that using a combined *P. vivax* population did not impact estimates of *P. falciparum* diversity, AMA1 diversity within the Mugil *P. vivax* population was also investigated. Diversity of AMA1 within the Mugil *P. vivax* population was similar to that of the combined Madang population, namely that diversity was high and exceeded that of the Madang (Mugil) *P. falciparum* population (Additional files 1 and 2). Hence, the differences observed were not due to stratified population effects.

**Figure 1:**
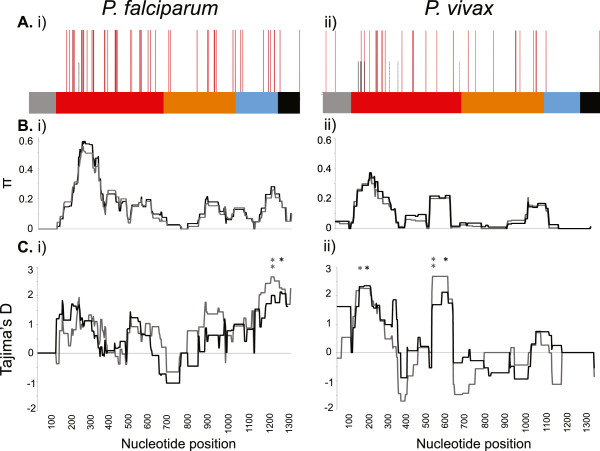
**Polymorphism and selection of AMA1 genes in *Plasmodium falciparum and Plasmodium vivax *populations of Papua New Guinea.** The following results are based on the total dataset of 76 *P. falciparum* and 102 *P. vivax* sequences **A)** Polymorphism: Schematic of the (i) *P. falciparum* and (ii) *P. vivax* genes encoding the AMA1 ectodomain, with all polymorphisms including non-synonymous (NS SNP, red lines), synonymous (SP SNP, black lines) and singleton (dashed red and black lines, respectively) sites shown. Location of residues is indicated by the colored panel along the top of the chart: signal sequence (grey), DI (red), DII (orange), DIII (blue), transmembrane region (black). **B)** Nucleotide diversity: Sliding window analysis showing nucleotide diversity (π values for (i) *Pfama1* and (ii) *Pvama1.* A window size of 100 bp and a step size of 3 bp were used. **C)** Natural selection. Sliding window calculation of Tajima’s D was performed for all (i) 76 *Pfama1* sequences and (ii) 102 *Pvama1* sequences (black = Madang; grey = Wosera). A window size of 100 and a step size of 3 were used. A single asterisk (black = Madang; grey = Wosera) indicates significant values for which p <0.05; and double asterisk indicates p <0.01.

Polymorphism was distributed unevenly among the three domains of AMA1 in both species. The number of SNPs, and consequently nucleotide diversity, peaked in DI for both *Pfama1* and *Pvama1* (Figures 1A and B; Table 1). Again, there were a greater number of SNPs in *Pfama1* DI compared to *Pvama1*, but a higher number of SP SNPs and increased allelic diversity resulted in higher overall diversity of *Pvama1* DI haplotypes (Table 1). In contrast, the diversity of *Pfama1* exceeded that of *Pvama1* in DII and DIII (Figure 1B; Table 1). For DII, the number of haplotypes in Madang was similar for both species, however in Wosera the number of *Pfama1* NS SNPs and haplotypes greatly exceeded that of *Pvama1* (Table 1). For DIII, there were seven NS SNPs and a peak in nucleotide diversity (π) for *Pfama1* with eight and nine haplotypes in the Wosera and Madang populations, respectively but only a single NS SNP and two haplotypes were detected for *Pvama1* in each population (Figure 1B; Table 1). No SP SNPs were detected in DII or DIII for either species.

### Evolution of *Plasmodium falciparum* and *Plasmodium vivax* AMA1 genes

To identify departures from neutral evolution, the Tajima’s D statistic was calculated using a sliding window approach along the length of the region encoding the AMA1 ectodomain for each population of both species. Although positive values of Tajima’s D, most likely due to balancing (immune) selection, were observed along the length of the ectodomain for *Pfama1*, highly significant positive values of Tajima’s D (*p* < 0.01) were observed in DIII (Figure 1C). This suggests that DIII is an important target of protective host immune responses within *Pfama1*.

For *Pvama1*, only DI deviated significantly from neutral expectations, suggesting that this region is a strong target of functional host immune responses (Figure 1C). Interestingly, although the results were similar for the Wosera and Madang *Pfama1* populations, differences between the Madang and Wosera *Pvama1* populations were observed. Whilst significantly positive values (*p* < 0.05) of Tajima’s D were observed within DI for both populations, negative values were also observed in DI for the Wosera population, which can indicate an excess of rare alleles at these sites consistent with purifying selection or a recent population expansion. The negative values of Tajima’s D did not reach significance (Figure 1C).

### Recombination and linkage disequilibrium (LD) in *Plasmodium falciparum* and *Plasmodium vivax* AMA1 genes

**Figure 2:**
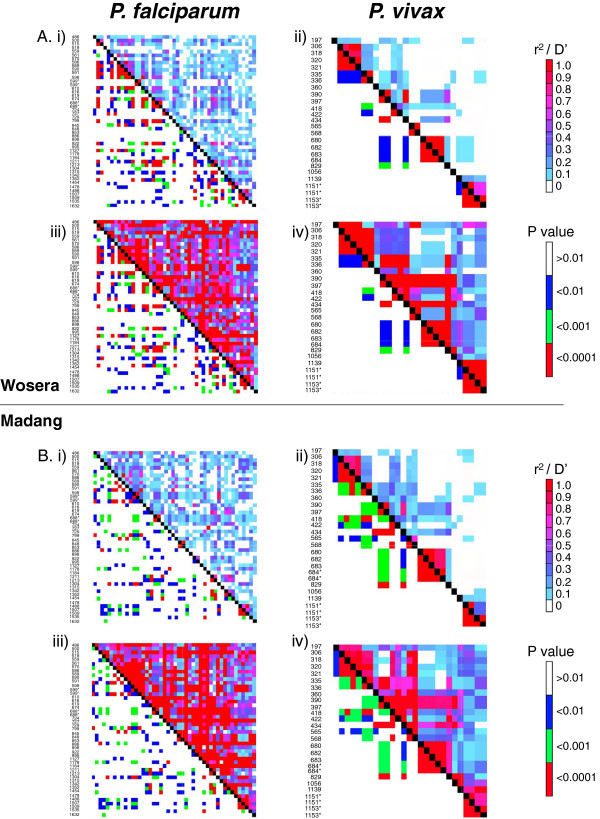
**Linkage disequilibrium in AMA1 genes of *Plasmodium falciparum and Plasmodium vivax *populations in Papua New Guinea.** The indices of linkage disequilibrium (LD), *r*^*2*^ (i and ii) and *D’* (iii and iv) were calculated for all AMA1 polymorphisms with a MAF ≥0.10 for **(A)** Wosera and **(B)** Madang populations. SNP position is shown on the Y axis and black squares represent self comparisons. Numbering is relative to the *Pfama1 3D7* reference sequence (GenBank accession no: XM_001347979.1) and the *Pvama1 Sal-1* reference sequence (GenBank accession no: AF063138), respectively. Coloured squares above the black diagonal represent values obtained for each pair of sites following *r*^*2*^ or *D'* calculations. Below the black diagonal line on each heat map, coloured squares reflect the significance value (*p*). An asterisk denotes tri-allelic SNPs that were split into two so that the major allele was analysed separately with each of the minor alleles. For interpretation, see the scale to the right of diagrams.

To explore how much genetic exchange occurs at the AMA1 locus in each species, the amount of recombination and LD were estimated. The recombination parameter, *R,* was higher for *P. vivax* compared to *P. falciparum*, and was higher in the Wosera compared to Madang for both species (Table 1). Linkage disequilibrium was then estimated between all pairs of ‘common’ polymorphic SP and NS SNPs (*Pfama1*, n = 45; *Pvama1* = 24). Significant (*p* < 0.0001) LD values between distant sites were observed for *P. falciparum*, whereas LD decayed with physical distance between *P. vivax* polymorphic sites (Figure 2). Together, these data demonstrate that the rate of recombination in *Pvama1* exceeds that of *Pfama1* in these populations across the region evaluated.

### Three-dimensional structural modeling of *Pf*AMA1 and *Pv*AMA1 polymorphisms

All residues found to be polymorphic were mapped to the respective three-dimensional model for *P. falciparum* and *P. vivax* AMA1. Previously, only *Pv*AMA1 polymorphisms predicted to be under balancing selection were mapped to the *P. vivax* three-dimensional model [51]. In the present study, all polymorphic loci were mapped, extending the previous study by including eight additional sites polymorphic amongst PNG *Pv*AMA1 sequences: M51, D133, A172, G253, R317, T359, K400 and Q484. In order to determine the proximity of the polymorphic residues to the ligand-binding cleft, the residues comprising the hydrophobic cleft [77] were also mapped.

**Figure 3:**
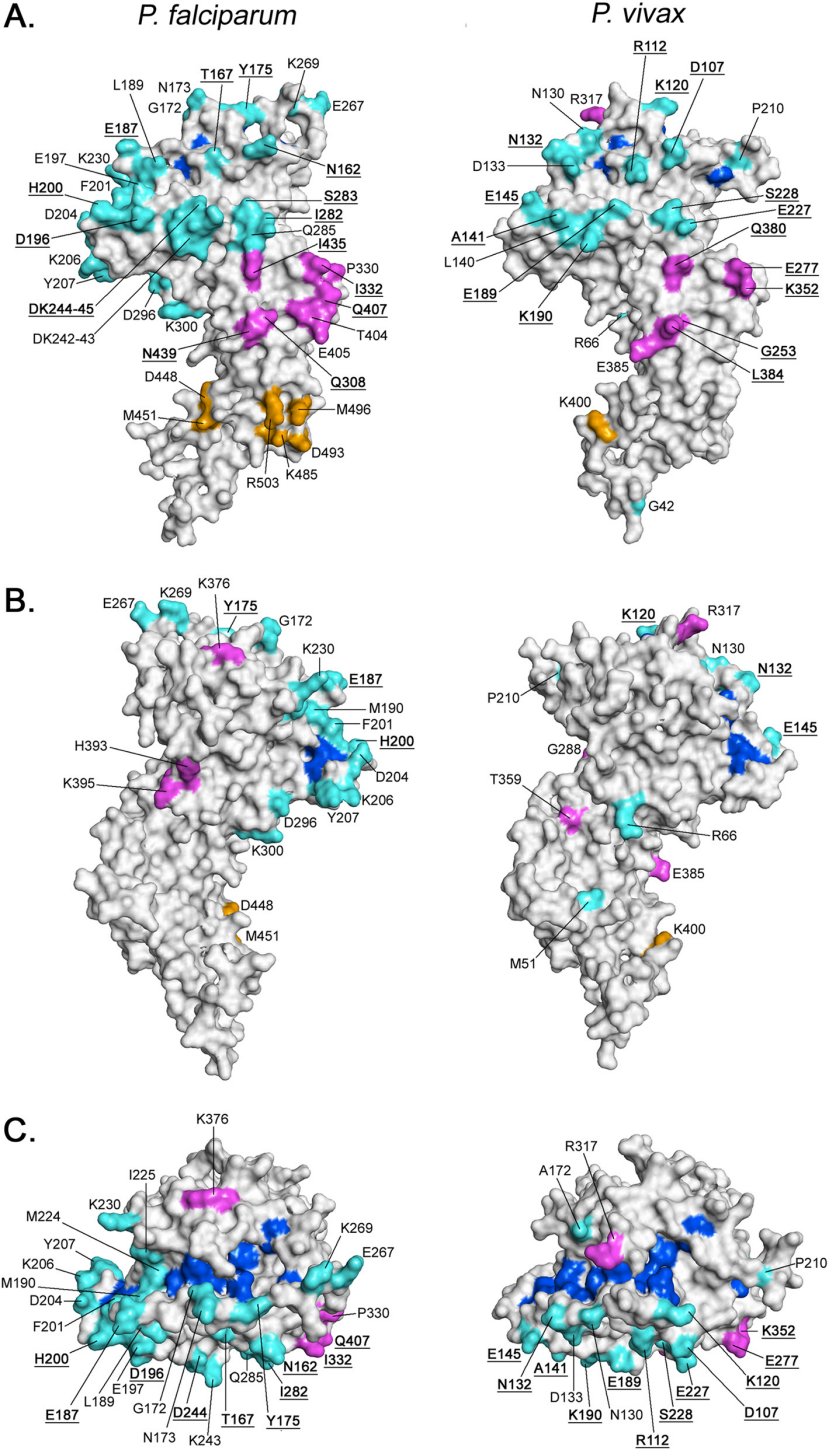
**Three-dimensional structural model of *Plasmodium falciparum and Plasmodium vivax *AMA1 polymorphisms. A)** Solvent-accessible surface representation of the ‘active face’ of the *Pf*AMA1 and *Pv*AMA1 three-dimensional (3D) models. Polymorphic residues are colored according to location: DI in cyan, DII in magenta, DIII in orange. Hydrophobic ligand binding cleft residues are shown in dark blue. Residues labeled with bold, underlined type are polymorphic in both *P. falciparum* and *P. vivax*. Residues of potential immunological relevance with a MAF ≥0.10 are indicated with an asterisk. **B)** Solvent-accessible surface representation of the ‘silent face’ of the *Pf*AMA1 and *Pv*AMA1 models. The hydrophobic cleft and polymorphic residues are shown, with coloring and labeling as described for panel A. **C)** Solvent-accessible surface representation of the *Pf*AMA1 and *Pv*AMA1 models showing a top-view of the hydrophobic binding cleft. Hydrophobic cleft and polymorphic residues are shown, with coloring and labeling as described for panel A. Note that for *Pf*AMA1, hydrophobic cleft residues M224 and M190 are polymorphic and colored cyan (not dark blue) as they are in DI.

All polymorphic residues (*Pf*AMA1, n = 47; *Pv*AMA1, n = 28) identified amongst PNG isolates that were located within the region used to generate the respective models (Additional file 3) mapped to solvent-exposed surfaces (Figure 3). Consistent with the findings of Bai *et al*. [33], a biased distribution of polymorphisms was observed for both species, with 41 (87%) *Pf*AMA1 polymorphic residues and 25 (89%) *Pv*AMA1 located on one face of the AMA1 molecule. For *Pf*AMA1, three polymorphic DII residues (K376T, H393R and K395R) were located on the opposing ‘silent’ face (Figure 3; Additional file 4). For *Pv*AMA1, two polymorphic signal sequence residues, M51I and R66K, and one DII residue, T359A, were located on the opposing ‘silent’ face (Figure 3; Additional file 5).

In both species, Tyr251, the hydrophobic cleft residue reported to be essential for RON2 binding [78] was strictly conserved (Additional file 3). Of the 15 orthologous sites that were polymorphic in both species, ten were located within DI and five within DII (Additional files 3, 4 and 5). Eight of those surrounding the hydrophobic binding cleft have been previously associated with antigenic escape for *Pf*AMA1 ([11,79], Additional files 3, 4 and 5).

Whilst the overall distribution of polymorphic residues was similar for both species, important differences with regards to species-specific polymorphisms surrounding and comprising the binding cleft were observed. Two hydrophobic binding cleft residues, M190I and M224I, were found to be polymorphic in 25 and 4% of PNG *Pf*AMA1 sequences, respectively (Figure 3; Additional files 3 and 4). In all sequences only one of these sites, but not both, was polymorphic. None of the *Pv*AMA1 hydrophobic cleft residues were found to be polymorphic. Additionally, all polymorphic *Pf*AMA1 residues observed amongst PNG sequences that were located proximal to the binding cleft have been associated previously with antigenic escape [11,79]. For *Pv*AMA1, with the exception of N130K, all polymorphic residues located proximal to the binding cleft also aligned with *Pf*AMA1 residues belonging to the c1 or c3 clusters associated with antigenic escape (Figure 3; Additional file 3). However R317Q, found in three (3%) PNG *Pv*AMA1 sequences, was located in DII and immediately proximal to the hydrophobic binding cleft (Additional file 5). The orthologous *Pf*AMA1 residue, Ala372, was strictly conserved.

### Relevance of *Pf*AMA1 and *Pv*AMA1 diversity to vaccine design

In order to compare and contrast the extent of *Pf*AMA1 and *Pv*AMA1 antigenic diversity within PNG, haplotypes were constructed using ‘common’ amino acid polymorphisms (MAF ≥0.10), as these polymorphisms are predicted to contribute the majority of antigenic diversity and are therefore relevant to vaccine design [51].

**Figure 4:**
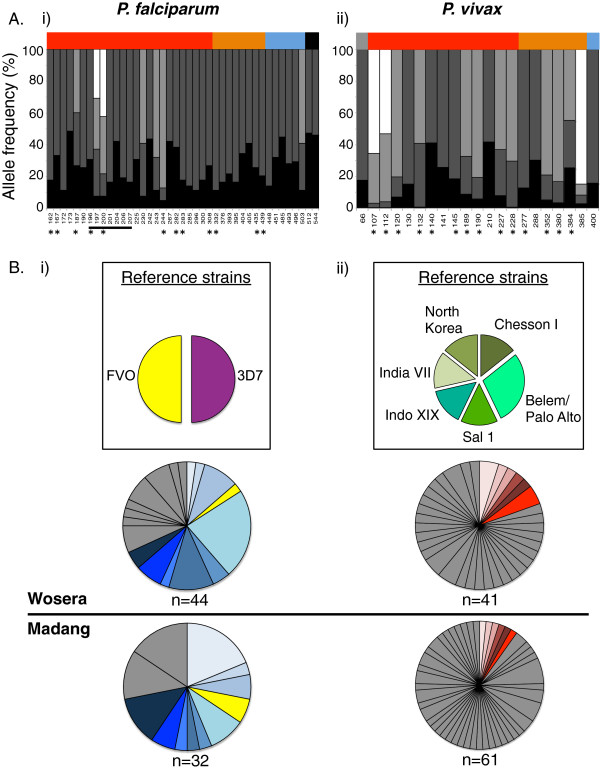
**Frequency of AMA1 polymorphisms and haplotypes in *Plasmodium falciparum and Plasmodium vivax *populations in Papua New Guinea. A)** Polymorphisms. The frequencies of common polymorphisms are shown for (i) *Pf*AMA1 (n = 41) and (ii) *Pv*AMA1 (n = 18). Location of residues is indicated by the colored panel along the top of the chart: signal sequence (grey), DI (red), DII (orange), DIII (blue), transmembrane region (black). Allele frequencies are indicated by the proportion of each bar shaded. Sites that are polymorphic in both species are indicated by an asterisk. Antigenic escape residues defined for *Pf*AMA1 (the “c1L” cluster) are indicated by the horizontal black line (ii). **B)** Haplotypes. Frequencies of haplotypes based on common polymorphisms for (i) *Pf*AMA1 (n = 21) and (ii) *Pv*AMA1 (n = 78). Coloured segments indicate shared haplotypes between the two populations and grey indicates those unique to one population. The size of the fragment reflects the relative frequency of the haplotype within the population. Reference strain haplotypes are colored to highlight presence/absence in the populations investigated. For *Pf*AMA1, only one haplotype was identical to a reference strain (*FVO*). No naturally circulating *Pv*AMA1 strains shared haplotypes with any of the reference strains analysed, and thus they are shown in grey. Sample size (n) and origin are indicated.

Of 48 *Pf*AMA1 polymorphic amino acid sites, 41 had a MAF, or combined MAF of ≥0.10 (Figure 4A). These ‘common’ sites included the highly polymorphic c1 and c1L cluster residues that have been previously associated with antigenic escape [79]. An additional *Pf*AMA1 polymorphic residue at the C-terminal end (position 544) was also included in the haplotype analysis but was not mapped to the *Pf*AMA1 model as it was located outside the region used to generate the crystal structure. Of the 41 ‘common’ *Pf*AMA1 polymorphisms, the majority (n = 34, 83%) were dimorphic. Five polymorphisms were tri-allelic (12%) and two were tetra-allelic (5%) (Figure 4A). The majority of common polymorphisms were clustered in DI (n = 25, 61%, Figure 4A).

Previously, PvAMA1 polymorphism amongst a population of global sequences was investigated [51]. Whilst it was reported that PNG PvAMA1 sequences were distinct from all others worldwide, as global diversity was the focus of the previous study, the mutations and combinations thereof unique to PNG were not investigated. In the present study, when compared to the 23 sites with a MAF ≥0.10 identified amongst the global *P. vivax* population, three sites (N132, P210, G288) had a MAF <0.10 and one site (R438) was invariant amongst PNG sequences. Residue D133, invariant amongst all other populations worldwide [51] had a MAF ≥0.10 amongst PNG sequences. Additionally, seven polymorphic sites with a MAF <0.10 amongst the global population were found to be invariant amongst PNG *Pv*AMA1 sequences: G117, D158, H193, V218, K368, V382, N445. Hence, although *Pv*AMA1 diversity was investigated previously, the residues and haplotypes analysed in the present study differ considerably on account of the unique polymorphisms and combinations thereof identified amongst PNG *Pv*AMA1 sequences.

Amongst PNG *Pv*AMA1 sequences, 28 sites were polymorphic however only 18 were common (Additional file 5), less than half the number detected for *Pf*AMA1. *Pv*AMA1 allelic diversity was however higher than of *Pf*AMA1 with a larger proportion of tri-allelic (*Pf*: n = 5, 12%; *Pv*: n = 5, 27%) sites (Figure 4A). None of the polymorphic *Pv*AMA1 sites were tetra-allelic.

Based on the 41 common *Pf*AMA1 polymorphisms, only 21 *Pf*AMA1 haplotypes were detected (Madang =12, Wosera =18). Despite less than half the number of common amino acid polymorphisms compared to *Pf*AMA1, there were 78 unique *Pv*AMA1 haplotypes (Madang = 48, Wosera = 36). Almost half (47.6%, n = 10) of the *Pf*AMA1 haplotypes were shared between Madang and Wosera, and four haplotypes were highly prevalent (frequency = 11.3 to 22.7%). Conversely, all 78 *Pv*AMA1 haplotypes were relatively rare (frequency <6.5%) with only 7.7% (n = 6) shared between Madang and Wosera (Figure 4B). This further demonstrates that in PNG, *Pf*AMA1 diversity is substantially lower than *Pv*AMA1 diversity.

Of direct relevance to vaccine design, the prevalence of alleles being used in vaccine development were then investigated. Whilst the *Pf*AMA1 vaccine allele, *FVO* was found in both populations, albeit at a low frequency (Madang = 6.2%, Wosera = 2.2%; Figure 3B), the *Pf*AMA1 vaccine allele 3D7 and none of the seven primate-adapted *Pv*AMA1 alleles used for vaccine development, (*Sal-1, Chesson I, Belem, India VII, Indonesia XIX, Palo Alto* and *North Korea*), were detected in either population (Figure 4B).

Polymorphism of residues located within the epitopes of invasion inhibitory antibodies is associated with immune escape [11,79,80]. All five of the *Pf*AMA1 c1 cluster residues that are essential for binding of the inhibitory antibody 1F9 were polymorphic (E197G/D/Q, H200D/L/R, F201L, D204N and I225N; Additional file 4) [77]. It has been reported that any substitution of the *Pf*AMA1 Glu197 completely abrogates 1F9 binding [77]. Only five of the 76 PNG *Pf*AMA1 sequences investigated (6.5%) had the reference allele at this residue (Additional file 4: Table S1). Six *Pf*AMA1 DII residues (Asp348, Lys351, Gln352, Phe385, Asp388 and Arg389) reported to be critical for binding of the 4G2 inhibitory antibody [81] were conserved amongst the *Pf*AMA1 PNG sequences analysed.

## Discussion

This is the first study of comparative *P. falciparum* and *P. vivax* genetic diversity to be conducted in a setting of high or similar co-endemicity, and the first study to directly compare the diversity of sympatric parasite populations in PNG. Additionally, presented here is the most comprehensive analysis of *P. falciparum* AMA1 diversity in PNG performed to date. Indices of recombination, haplotype number and diversity for *Pv*AMA1 exceeded those of *Pf*AMA1 in both geographic areas investigated. Consistent with previous observations following analysis of neutral molecular markers [53-56], it is likely that the effective population size of *P. vivax* exceeds that of *P. falciparum* within PNG.

For each species, diversity was similar in both geographic areas investigated despite the fact that Wosera had a lower prevalence of *P. vivax* than Madang [54] and a highly variable prevalence of *P. falciparum* among the different villages [55]. In the years prior to sample collection, increased access to treatment and the widespread roll-out and use of insecticide-treated bed nets (ITNs) resulted in a significant decline in the prevalence of both *P. falciparum* and *P. vivax* infection in the Wosera [82]. However, in the same geographic area in the year 2000, 27 *Pf*AMA1 DI haplotypes were identified from 168 samples [35] compared to the 18 haplotypes from only 44 samples collected in 2005 (this study), suggesting that reductions in prevalence have had a limited impact on *Pfama1* diversity. Reduced malaria parasite prevalence is a clear indicator that the control measures implemented in the Wosera have been effective. However, further substantial decreases in the prevalence of both species are required within this region to impact parasite antigenic diversity, which has long been a barrier to development of a successful malaria vaccine [22,83,84].

Whilst the overall distribution of polymorphic residues was similar between the species, species-specific differences with regard to the number of polymorphic residues, levels of synonymous polymorphism and substitution of specific AMA1 residues were observed. Consistent with previous observations, the level of synonymous polymorphism amongst *Pvama1* sequences exceeded that of *Pfama1*[48]. Although higher than *Pfama1*, it has been suggested that synonymous polymorphism in *Pvama1* may not be neutral as the level of *Pvama1* synonymous polymorphism is greatly reduced in comparison to another *P. vivax* merozoite antigen gene, merozoite surface antigen 1 (*Pvmsp1*) [52]. In addition, the overall number of polymorphic residues in *P. falciparum* was almost double that of *P. vivax*. Amino acid polymorphisms within malarial antigens are typically clustered within sequences coding for B- and T-cell epitopes, hence are associated with immune evasion [85]. Indeed, AMA1 substitutions of potential clinical and functional importance were found in PNG parasites. These included *Pf*AMA1 K230Q/E, which has previously been associated with clinical malaria in Kenyan children and is located at the periphery of the interface between AMA1 and growth-inhibitory antibody 1F9 [36]. Located within or proximal to the hydrophobic binding cleft, the *Pf*AMA1 M190I and M224I, and *Pv*AMA1 R317Q substitutions were also observed. The latter two substitutions have not been previously described, and may play a role in immune evasion, or have functional consequences for binding to RON2 [78]. However, it has been reported that not all genetic diversity within *Pf*AMA1 is antigenically relevant and that indeed it may be overcome with careful selection of a few divergent alleles [80]. Whereas the majority of polymorphic *Pf*AMA1 residues were bi-allelic, 27% of the *Pv*AMA1 polymorphic residues had three variants, contributing to the higher overall diversity. Whether the same diversity-covering approach can be used to overcome *Pv*AMA1 diversity remains unknown, and studies to identify the specific *Pv*AMA1 residues associated with immune evasion must be performed.

Furthermore, although AMA1 DI was clearly under balancing selection in both species, the strongest signatures of immune selection were detected in DI for *P. vivax*, and DIII for *P. falciparum,* suggesting that the dominant immune targets may differ between the species. Analysis of 64 *Pvama1* sequences from Sri Lanka demonstrated that *Pv*AMA1 DII is also under selection [40]. However, the results of the present study are analogous to those obtained for 372 global *Pv*AMA1 sequences, the largest and most comprehensive analysis of worldwide *Pv*AMA1 diversity performed to date, and suggest that DI is an immunodominant region of *Pv*AMA1 [51]. That nucleotide diversity and signatures of immune selection for *Pf*AMA1 are highest within DI and DIII, has also been reported following analysis of African *P. falciparum* sequences [36,86]. It has been proposed that strong signatures of selection within *Pf*AMA1 DIII may be due to T-cell responses against allele-specific epitopes [36,87,88]. However, LD between polymorphic DI and DIII residues was also observed amongst *Pf*AMA1, but not *Pv*AMA1, sequences. Previously, LD was reported to be absent among putatively neutral microsatellites in the same sample set [53]. Hence, the LD between *Pf*AMA1 DI and DIII residues might be the result of conformational association between residues comprising important domain-spanning epitopes [36] or alternatively, compensatory mutations to preserve protein function. However, as none of the linked residues co-localized when mapped to the *Pf*AMA1 3D structural model, this is unlikely.

Differences between the two malaria species with regards to levels of polymorphism, diversity, LD and rates of recombination might instead reflect differences in population size and potentially, divergent evolutionary histories of *P. falciparum* and *P. vivax* within PNG. Recombination accounts for the majority of the diversity observed within malarial antigens [85]. It occurs much more frequently than point mutations resulting in amino acid changes, and can change several nucleotides at once in a single event [85]. As recombination results in production of new variants by combining existing types found within a population, effective population size is a key determinant of population genetic diversity [76,89]. Genome-wide sequencing of a small panel of geographically diverse isolates has demonstrated that the worldwide genetic diversity and effective population size of *P. falciparum* is greatly reduced compared to *P. vivax*, due to at least one major population bottleneck and multiple drug-induced selective-sweeps [8]. Despite also experiencing strong drug pressure, the greater stability of the larger, more diverse and ancient global *P. vivax* population can be attributed to the latent hypnozoite reservoir and more rapid rate of gametogenesis [8]. During past malaria elimination attempts in PNG, large-scale malaria control programs carried out from the 1950s-70s may have initially had a greater impact on *P. falciparum* until the emergence of chloroquine-resistant strains [90,91]. In the 1980s, chloroquine-resistant *P. vivax* also emerged in PNG [92,93], although the drug pressure on this parasite is not expected to be as strong as that on *P. falciparum* due to the lower frequency of clinical cases and anti-malarial treatment. Analysis of neutral microsatellite markers has shown that *P. falciparum* populations of Madang and Wosera are genetically differentiated [53], however very limited population structure is observed in *Pfama1*. This might be the result of past reduction in effective population size (e g, a bottleneck), after which common AMA1 haplotypes would be maintained by balancing selection while differentiation at neutral markers would increase through genetic drift. In contrast, very weak *P. vivax* population structure [56] and detection of a high number of extremely diverse, low frequency *Pv*AMA1 haplotypes is consistent with a stable and large effective population size, high levels of recombination and high gene flow between areas. Divergent values of Tajima’s D for *Pvama1* sequences from the Wosera and not Madang potentially suggests that the environmental or selective pressures acting on the Madang and Wosera *P. vivax* populations may not be the same (i.e. local adaptation). Combined, it is therefore likely that in PNG, *Pvama1* diversity exceeds that of *Pfama1* on account of a larger, more frequently recombining and more ancient *P. vivax* population compared to *P. falciparum*. However, as microsatellites are highly polymorphic [12,59] and AMA1 is under selection in both species [36,51], it will be necessary to analyse more slowly evolving loci, such as mitochondrial DNA in order to clarify the evolutionary histories of *P. falciparum* and *P. vivax* in PNG.

## Conclusions

It is likely that the diversity of *Pv*AMA1 far exceeds that of *Pf*AMA1 in a highly endemic region of PNG because of the underlying parasite population biology [53,94]. Diversity was extremely high in both species and in both populations investigated, despite recent reductions in prevalence in one area (Wosera). Hence, far greater reductions in prevalence of both species will be required to impact the diversity of *P. falciparum* and *P. vivax* AMA1. Differing patterns of balancing selection suggest that dominant immune targets may differ between the two species, which is important knowledge for development of AMA1-based vaccines. Furthermore, vaccine haplotypes for both species were found to circulate at very low frequencies if at all, in PNG. As inclusion of alleles not representative of a given population may result in poor vaccine efficacy [80], this important observation suggests that vaccines based on reference strains might have limited efficacy in this region. The results of this study highlight differences in the population biology and evolution of *P. falciparum* and *P. vivax* in PNG, and have important implications for the design of AMA1-based vaccines.

## Competing interests

The authors declare that they have no competing interests.

## Authors’ contributions

AEB and JCR conceived and designed the experiments. AA and JW performed the experiments. AA, AEB and PAR analysed the data. IM and PAR contributed reagents/materials/analysis tools. AA, AEB, JCR, and PAR wrote the paper. PS provided logistical support. All authors read and approved the final manuscript.

## Supplementary Material

Additional file 1 **Frequency of AMA1 polymorphisms and haplotypes in the *Plasmodium falciparum *and *Plasmodium vivax *Madang and Mugil populations of Papua New Guinea.** i) Haplotypes. Frequencies of haplotypes based on common polymorphisms for (A) *Pv*AMA1 Madang (n = 18), (B) *Pv*AMA1 Mugil (n = 18) and (C) *Pf*AMA1 (n = 41). Coloured segments indicate shared haplotypes between the Wosera and Madang/Mugil populations for each species. Grey indicates haplotypes unique to the specified population. For *Pf*AMA1, only one haplotype was identical to a reference strain (yellow: *FVO*). No naturally circulating *Pv*AMA1 strains shared haplotypes with any of the reference strains analysed. Sample size (n) and origin are indicated. ii) Polymorphisms. The frequencies of common polymorphisms are shown for (A) *Pv*AMA1 Madang (n = 18), (B) *Pv*AMA1 Mugil (n = 18) and (C) *Pf*AMA1 (n = 41). Location of residues is indicated by the colored panel along the top of the chart: signal sequence (grey), DI (red), DII (orange), DIII (blue), transmembrane region (black). Allele frequencies are indicated by the proportion of each bar shaded. Click here for file

Additional file 2 **Estimates of AMA1 genetic diversity for *Plasmodium falciparum *and *Plasmodium vivax* within Madang and Mugil.** Click here for file

Additional file 3 **Protein sequence alignment showing regions of *Plasmodium falciparum *and *Plasmodium vivax *AMA1 used to generate three-dimensional models.** The *P. falciparum* 3D7 (GenBank accession no: XM_001347979) and *P. vivax Sal-1* (GenBank accession no: AF063138) reference sequences were aligned using MEGA version 5.0 [65]. Numbers indicate the position of residues relative to those of the *P. falciparum* sequence. Gaps are indicated by dashes. Red bold type indicates residues observed to be polymorphic in both species; black bold type indicates residues polymorphic in either *P. falciparum* or *P. vivax*. The domain boundaries are demarcated by vertical lines, as indicated. Boxes indicate the positions of antigenic *P. falciparum* amino acid clusters, c1-3 [79]; grey shading indicates antigenic escape residues in the c1L cluster [11]. Click here for file

Additional file 4 **Summary of polymorphisms in *Pf*AMA1 sequences from Papua New Guinea.** All 47 residues polymorphic amongst PNG *Pf*AMA1 sequences are summarized here. The amino acid position (relative to reference isolate *3D7*), the *3D7* allele, the variant allele(s) observed, the number of sequences identical to *3D7* at each polymorphic site and the number of sequences containing the variant allele(s) at each polymorphic site are listed. Sites identical to the *3D7* sequence are indicated by a dot. The 41 polymorphisms with a minor allele frequency (MAF) ≥0.10 are shown in bold. Sites polymorphic in both species are highlighted in red. Click here for file

Additional file 5 **Summary of polymorphisms in *Pv*AMA1 sequences from Papua New Guinea.** All 28 residues polymorphic amongst PNG *Pv*AMA1 sequences are summarized here. The amino acid position (relative to reference isolate *Sal-1*), the *Sal-1* allele, the variant allele(s) observed, the number of sequences identical to *Sal-1* at each polymorphic site and the number of sequences containing the variant allele(s) at each polymorphic site are listed. Sites identical to the *Sal-1* sequence are indicated by a dot. The 18 polymorphisms with a minor allele frequency (MAF) ≥0.10 are shown in bold. Sites polymorphic in both species are highlighted in red. Click here for file
