# Supplementary figures and images for: Distinct patterns of diversity, population structure and evolution in the AMA1 genes of sympatric Plasmodium falciparum and Plasmodium vivax populations of Papua New Guinea from an area of similarly high transmission
Source: Malar J. 2014 Jun 14;13:233. doi: 10.1186/1475-2875-13-233 (PMC4085730; doi:10.1186/1475-2875-13-233)

**A) *P. vivax*: Madang**

i.

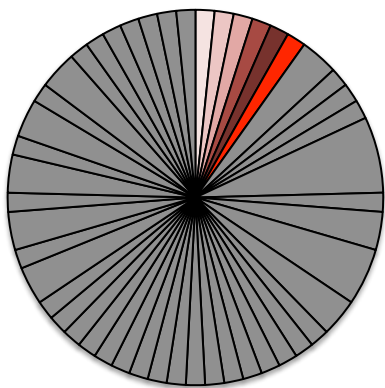

n=61

ii.

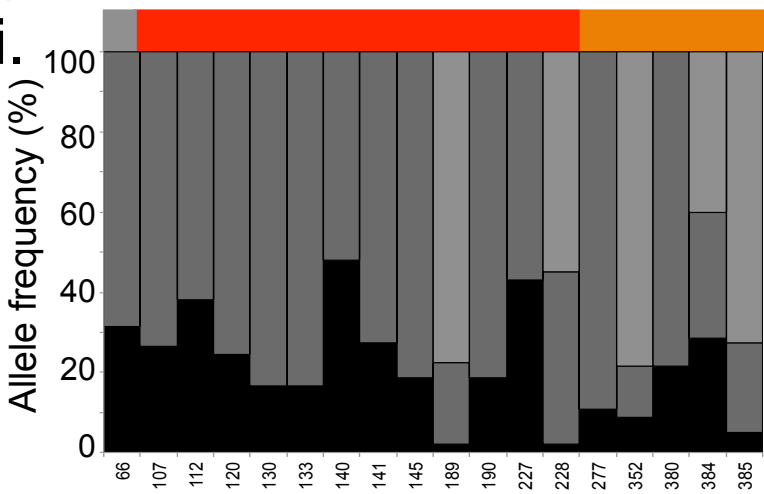

**B) *P. vivax*: Mugil**

i.

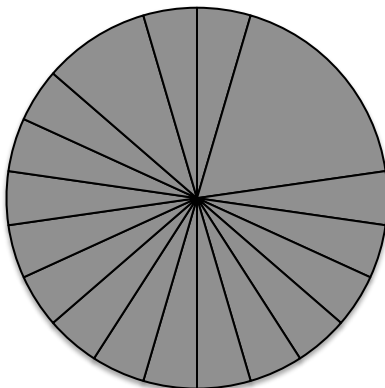

n=22

ii.

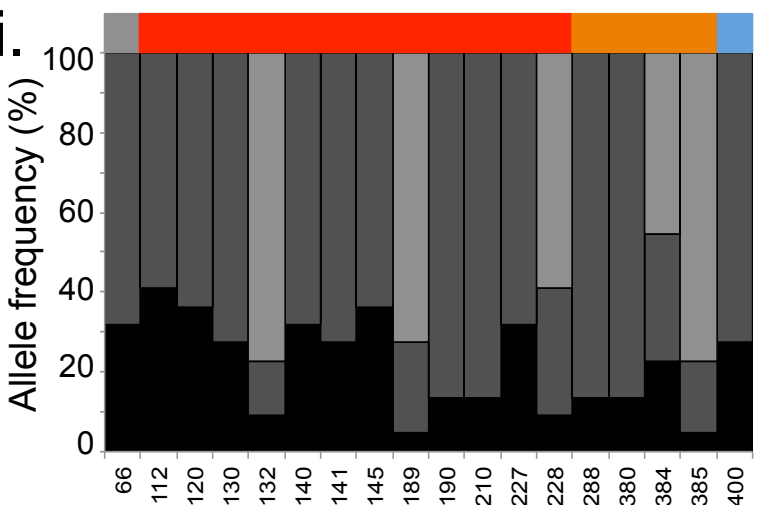

**C) *P. falciparum*: Madang (Mugil)**

i.

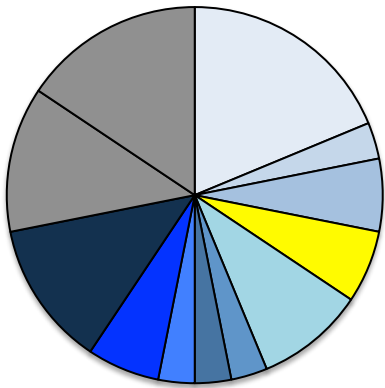

n=32

ii.

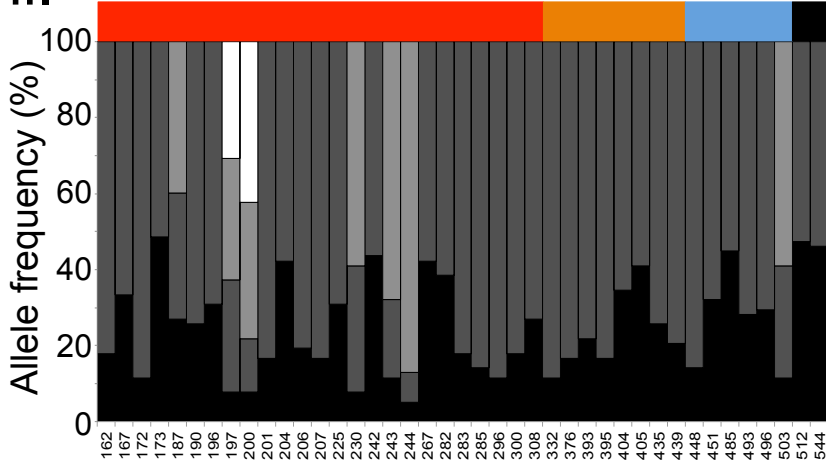

Supplement: Additional file 1 — Frequency of AMA1 polymorphisms and haplotypes in the Plasmodium falciparum and Plasmodium vivax Madang and Mugil populations of Papua New Guinea. i) Haplotypes. Frequencies of haplotypes based on common polymorphisms for (A) PvAMA1 Madang (n = 18), (B) PvAMA1 Mugil (n = 18) and (C) PfAMA1 (n = 41). Coloured segments indicate shared haplotypes between the Wosera and Madang/Mugil populations for each species. Grey indicates haplotypes unique to the specified population. For PfAMA1, only one haplotype was identical to a reference strain (yellow: FVO). No naturally circulating PvAMA1 strains shared haplotypes with any of the reference strains analysed. Sample size (n) and origin are indicated. ii) Polymorphisms. The frequencies of common polymorphisms are shown for (A) PvAMA1 Madang (n = 18), (B) PvAMA1 Mugil (n = 18) and (C) PfAMA1 (n = 41). Location of residues is indicated by the colored panel along the top of the chart: signal sequence (grey), DI (red), DII (orange), DIII (blue), transmembrane region (black). Allele frequencies are indicated by the proportion of each bar shaded. [file 1475-2875-13-233-S1.pdf]
