# Supplementary material for: Distinct patterns of diversity, population structure and evolution in the AMA1 genes of sympatric Plasmodium falciparum and Plasmodium vivax populations of Papua New Guinea from an area of similarly high transmission
Source: Malar J. 2014 Jun 14;13:233. doi: 10.1186/1475-2875-13-233 (PMC4085730; doi:10.1186/1475-2875-13-233)
Supplement: Additional file 2 — Estimates of AMA1 genetic diversity for Plasmodium falciparum and Plasmodium vivax within Madang and Mugil. [file 1475-2875-13-233-S2.docx]

**Additional file 2: Estimates of AMA1 genetic diversity for *P. falciparum* and *P. vivax* within Madang and Mugil**

|  | **Whole ectodomain** | | | | | | | | |
| --- | --- | --- | --- | --- | --- | --- | --- | --- | --- |
|  | *n* | *S* | ∏  (x 10^-3^) | NS | SP | *h* | *Hd* | *R_s_* | *R* |
| ***P. falciparum*** |  |  |  |  |  |  |  |  |  |
| Madang (Mugil) | 32 | 52 | 13.4 | 47^a^ | 1^a^ | 12 | 0.91 | 12 | 36.6 |
| ***P. vivax*** |  |  |  |  |  |  |  |  |  |
| Madang (inclu. Mugil) | 61 | 36 | 8.5 | 29^b^ | 4^b^ | 50 | 0.99 | 45 | 66.2 |
| Mugil | 22 | 35 | 8.9 | 30^c^ | 4^c^ | 19 | 0.97 | 19 | 68.4 |

*n*=number of samples; *S*=number of polymorphic sites; ∏=nucleotide diversity; NS= number of non-synonymous single nucleotide polymorphisms (SNPs); SP=number of synonymous SNPs; *h*=number of haplotypes; *Hd*=haplotype diversity; *R_s_*: allelic richness; *R*: recombination parameter

^a^ Complex codons not analysed by DnaSP software: 2 codons (292, 293, 294) (301, 302, 303)

^b^ Complex codon not analysed by DnaSP software: 1 codon (550, 551, 552)

^c^ Complex codon not analysed by DnaSP software: 1 codon, 1 site affected (204)
