# Supplementary material for: Distinct patterns of diversity, population structure and evolution in the AMA1 genes of sympatric Plasmodium falciparum and Plasmodium vivax populations of Papua New Guinea from an area of similarly high transmission
Source: Malar J. 2014 Jun 14;13:233. doi: 10.1186/1475-2875-13-233 (PMC4085730; doi:10.1186/1475-2875-13-233)
Supplement: Additional file 3 — Protein sequence alignment showing regions of Plasmodium falciparum and Plasmodium vivax AMA1 used to generate three-dimensional models. The P. falciparum 3D7 (GenBank accession no: XM_001347979) and P. vivax Sal-1 (GenBank accession no: AF063138) reference sequences were aligned using MEGA version 5.0 [65]. Numbers indicate the position of residues relative to those of the P. falciparum sequence. Gaps are indicated by dashes. Red bold type indicates residues observed to be polymorphic in both species; black bold type indicates residues polymorphic in either P. falciparum or P. vivax. The domain boundaries are demarcated by vertical lines, as indicated. Boxes indicate the positions of antigenic P. falciparum amino acid clusters, c1-3 [79]; grey shading indicates antigenic escape residues in the c1L cluster [11]. [file 1475-2875-13-233-S3.docx]

DI

....|....| ....|....| ....|....| ....|....| ....|....| ....|....| ....|....| ....|....|

100 110 120 130 140 150 160 170

*P. falciparum* (3D7) SIEIVERSNY MGNPWTEYMA KYDIEEVHGS GIRVDLGEDA EVAGTQYRLP SGKCPVFGKG IIIENS**N**TTF L**T**PVAT**GN**Q**Y**

*P. vivax* (Sal-1) K**G**PTVERSTR **M**SNPWKAFME KYDIE**R**THSS GVRVDLGEDA EVENAKYRIP AGRCPVFGKG IVIENS**D**VSF L**R**PVATGDQ**K**

**c3**

....|....| ....|....| ....|....| ....|....| ....|....| ....|....| ....|....| ....|....|

180 190 200 210 220 230 240 250

*P. falciparum* (3D7) LKDGGFAFPP T**E**P**LM**SPMTL **DE**MR**HF**YK**D**N **KY**VKNLDELT LCSRHAGN**MI** PDND**K**NSNYK YPAVYD**DKDK** KCHILYIAAQ

*P. vivax* (Sal-1) LKDGGFAFP**N** A**ND**HISPMT**L A**NLK**E**RYKDN VEMMKLNDIA LCRTHAASFV M**A**GDQNSSYR HPAVYDEK**EK** TCHMLYLSAQ

**c1**

**c1L**

DII

**c2**

**c1**

....|....| ....|....| ....|....| ....|....| ....|....| ....|....| ....|....| ....|....|

260 270 280 290 300 310 320 330

*P. falciparum* (3D7) ENNGPRYCNK D**E**S**K**RNS**M**FC FRPAKD**IS**F**Q** NYTYLSKNVV **D**NWE**K**VCPRK NL**Q**NAKFGLW VDGNCEDIPH VNEF**P**A**I**DLF

*P. vivax* (Sal-1) ENMGPRYCS**P** DAQNRDAVFC FKPDKN**ES**FE NLVYLSKNVR NDWDKKCPRK NL**G**NAKFGLW VDGNCEEIPY VKEVEA**E**DLR

**c3 c3**

**c2**

....|....| ....|....| ....|....| ....|....| ....|....| ....|....| ....|....| ....|....|

340 350 360 370 380 390 400 410

*P. falciparum* (3D7) ECNKLVFELS ASDQPKQYEQ HLTDYEKIKE GFKNKNASMI **K**SAFLPTGAF KADRYKS**H**G**K** GYNWGNYN**TE** T**Q**KCEIFNVK

*P. vivax* (Sal-1) ECNRIVF**G**AS ASDQPTQYEE EMTDYQKIQQ GFRQNN**R**EMI KSAFLPVGAF NSDNFKSKGR GFNWANFDSV K**K**KCYIFN**T**K

DIII

....|....| ....|....| ....|....| ....|....| ....|....| ....|....| ....|....| ....|....|

420 430 440 450 460 470 480 490

*P. falciparum* (3D7) PTCLINNSSY IATTALSHP**I** EVE**N**NFPCSL YK**D**EI**M**KEIE RESKRIKLND NDDEGNKKI IAPRIFISDD**K** DSLKCPC**D**PE

*P. vivax* (Sal-1) PTCLINDKNF IATTALSHP**Q** EVD**LE**FPCSI YKDEIEREI**K** KQSRNMNLYS VDGER---I VLPRIFISNDK ESIKCPCEPE

....|....| ....|....| ....|....| ....|..

500 510 520 530

*P. falciparum* (3D7) **M**VSNSTC**R**FF VCKCVE**R**RAE VTSNNEVVVK EEYKDEY

*P. vivax* (Sal-1) RISNSTCNFY VCNCVEKRAE IKENNQVVIK EEFRDYY
